# Dance as an Adjunct Therapy for Neurological Rehabilitation – Creative Enrichment for Recovery (DAN-CER): Program Design and Protocol for a Mixed Methods Pilot to Assess Feasibility and Acceptability

**DOI:** 10.2196/69452

**Published:** 2025-08-26

**Authors:** Danielle Pretty, Michael Norwood, Tamara Ownsworth, Kelly Dungey, Susan Jones, Zara Gomes, Kelly Clanchy, Elizabeth Kendall

**Affiliations:** 1 School of Allied Health, Sport and Social Work Griffith University Gold Coast Australia; 2 The Hopkins Centre Griffith University Meadowbrook Australia; 3 School of Applied Psychology Griffith University Mt Gravatt Australia; 4 Neurosciences Rehabilitation Unit Gold Coast University Hospital Gold Coast Australia; 5 Van Norton Li Community Health Institute Queensland Ballet Brisbane Australia

**Keywords:** acquired brain injury, adaptive dance, adjunct therapy, dance, feasibility, neurorehabilitation

## Abstract

**Background:**

Dance is a novel recreational activity that may improve psychosocial outcomes in inpatient neurological rehabilitation; however, adapted dance programs in neurological rehabilitation settings are still emerging.

**Objective:**

This paper describes the co-design process undertaken to develop an adapted dance program for use in neurological rehabilitation. It also presents a study protocol aimed at evaluating the program’s feasibility, acceptability, and preliminary efficacy in a subacute hospital setting.

**Methods:**

A 3-phase co-design approach was used to develop the Dance as an Adjunct Therapy for Neurological Rehabilitation – Creative Enrichment for Recovery (DAN-CER) program and protocol, including knowledge seeking, seeking expert input, and refining. Information sources included a literature review, stakeholder meetings, workshops, and focus groups with clinicians and patients. This study has approval from two ethics committees (HREC/2023/QGC/99631 and GU 2023/813).

**Results:**

We undertook 4 workshops with Queensland Ballet, and 2 focus groups were undertaken with staff and neuroscience ward patients. The resultant program was mapped to the Template for Intervention Description and Replication (TIDieR) checklist. A mixed methods design was selected to evaluate the program. Primary outcomes are the feasibility and acceptability of the adapted dance program with data on accrual and attendance collected weekly. Semistructured interviews with patients and staff were conducted postintervention. The secondary outcome is the efficacy of DAN-CER for improving well-being and affect, with impact on fatigue monitored. Adapted dance classes and data collection assessments began in late October 2024, and data collection was completed in late February 2025. At the time of manuscript submission, 14 participants had been recruited. Interviews have been transcribed, and preliminary coding is underway; findings are expected to be submitted for publication in December 2025.

**Conclusions:**

The rigor of the multiphase co-design process enabled the development of an adapted dance intervention capable of accommodating the physical, cognitive, and communication challenges of neurorehabilitation ward patients. The proposed mixed methods protocol will enable multidimensional evaluation of the adapted dance program.

**International Registered Report Identifier (IRRID):**

DERR1-10.2196/69452

## Introduction

### Recovery From Brain Injury

After surviving a traumatic brain injury (TBI) or stroke, 35% to 44% of people will require inpatient neurological rehabilitation [1,2]. Many patients during this time report experiencing social dysfunction (eg, reduced ability to function socially and feelings of social isolation) and psychological distress, such as anxiety [3]. Although there are biological (eg, inflammation and disrupted neurocircuitry between the prefrontal cortex and the limbic system) [4,5] and social reasons (eg, role change) for this, the inpatient environment may also be a contributing factor. Specifically, one limitation of brain injury rehabilitation settings is the predominant focus of impairment-driven rehabilitation, delivered in risk-adverse environments that optimize efficiency [6]. Although for good reason, this can be at the expense of elements necessary to support a positive patient experience such as positive distractions [6] and social contact [7]. Further, when not completing intensive rehabilitation, inpatients are often exposed to large amounts of passive, nonstimulating activities such as watching television [8,9], highlighting the need for alternative stimulating experiences in neurological rehabilitation [10].

In the hope of improving patient well-being and therapeutic outcomes, there is now a push to enrich the neurological rehabilitation environment [11]. “Environmental enrichment” can be defined as activities designed to facilitate sensorimotor, cognitive, and social activity by the provision of a stimulating environment [12]. Applying this definition to neurological rehabilitation, such activities should be interesting or engaging to encourage voluntary participation [13].

One environmental enrichment activity suitable for inpatient brain injury recovery is *creative arts*. Using *creative arts,* group-based activities offer two distinct advantages to traditional group therapy in the neurological environment. Although verbal psychotherapy relies on dialogue as the primary mode of communication [14], creative arts instead provide a low-verbal alternative for emotional expression [15,16]. Additionally, creative arts approaches facilitate communication in a unique way, encouraging authentic, natural interactions within the group, which can offer a new way to connect among participants [17] and a potential avenue for peer support [18].

Adapted dance is a novel approach to a group-based creative arts environmental enrichment activity for use in inpatient neurological rehabilitation. Although all creative arts activities aim to foster self-expression and creativity [18], dance also uniquely engages multiple sensory, cognitive, and motor pathways [19] and is typically perceived as a meaningful activity that fosters a sense of agency [20]. Further, certain elements of dance can be strategically used as a pillar in the program to foster a sense of belonging. Specifically, the synchronized movement and social coordination fundamental in group dance have been shown to enhance social connection, elevate mood, and promote pro-social behaviors [21,22]. Together, the multisensory and social elements of dance make it a prime candidate for an enrichment activity.

There is sound empirical support that dance has positive psychosocial benefits for individuals experiencing an array of health conditions. Reviews highlight the positive psychosocial impacts of dance across various clinical populations, including individuals with affective disorders, Parkinson disease, schizophrenia [23], physical disability [24], or physical and mental illness [25] as well as older adults [26]. Although the psychosocial impact has been understudied in people with brain injury, the limited existing research indicates social benefits of dance in both the hospital [27-29] and community [30] settings. However, further research is warranted to explore the potential of dance for enhancing well-being in individuals with brain injury.

### Adapted Dance in Neurological Rehabilitation

To systematically evaluate adapted dance in neurological rehabilitation settings, it is essential to take an evidence-based approach to program design and use standardized reporting in publications. Specific methodologies for designing adapted dance programs tailored to neurological rehabilitation are emerging [31]. However, Fortin [32] argues that, across many dance health programs, more research has focused on the outcomes of dance rather than the program itself. Although dance movement therapy is a common therapeutic approach, it can include a variety of components. When tested in a neurologically healthy population, it was found that some elements should be used cautiously due to their negative effect [33]. However, detailed descriptions of the components included in adapted dance programs are rarely reported. For these reasons, Beaudry et al [34] advocated for consistent use of a standardized checklist by dance health program designers to advance the field. One such example is the Template for Intervention Description and Replication (TIDieR) checklist [35], which helps provide a comprehensive description of an intervention facilitating future replication.

Acceptability research is also essential for programs such as adapted dance in neurological rehabilitation. Acceptability in health care interventions refers to the extent to which the intervention is considered appropriate by either the people delivering or receiving the intervention [36]. Examples of acceptability constructs are dropout rates, reasons for discontinuation, side effects, ratings of satisfaction, and feelings toward and perceptions of the intervention [36]. Although several dance studies to date have trialed adaptations, a scoping review found that, in many studies, the acceptability of these programs was not assessed by end users (D Pretty et al, unpublished data, August 2024). However, reviews of community dance programs and other complementary rehabilitation programs have highlighted that exploring feasibility and acceptability is a critical phase prior to formal evaluation and implementation [30,37].

Reviews indicate that few adapted dance programs in neurological rehabilitation use a co-design approach. In the review by Kipnis et al [30], only one-half of the programs used movements recommended by therapists. This is similar in adapted dance for people with TBI. Our review found that adapted dance programs were rarely developed with clinician input or through a co-design approach that involved individuals with lived experience of a brain injury (D Pretty et al, unpublished data, August 2024). Collaborative involvement of clinicians and patients in the design process can help to ensure the final program meets safety requirements while also being enjoyable and engaging. Multidisciplinary involvement may also help stakeholders build trust in the program, which will assist in the program running.

In summary, the acceptability of adapted dance programs in neurological rehabilitation has not been thoroughly assessed. For adapted dance programs to align with other neurological rehabilitation practices, the design process should engage end users and be multidisciplinary, and the program should be reported in a systematic fashion.

### Current Limitations in Neurological Rehabilitation Assessment

A key challenge evident in the neurological rehabilitation literature is understanding the individual differences in outcomes from interventions [38,39] and the variability from day to day (see [40]). Repeated measurements of psychosocial function, such as in a single case design approach, can reduce the threats to internal validity posed by this variability. This approach allows researchers to better understand the impact of adapted dance at an individual level, which is particularly important given the heterogeneity of brain injuries [41].

An additional priority for patient safety and ethical conduct in neurological rehabilitation research is the adequate monitoring of fatigue throughout adapted dance. Various researchers have identified fatigue as a potential barrier to participation [42] and a potential adverse effect from adapted dance [27,29]. However, fatigue has not been systematically examined in adapted dance studies. Fatigue is a multifaceted issue, including both mental and physical components [43] that must be addressed and monitored throughout the program to ensure patient safety.

### The Need for a Mixed Methods Feasibility and Acceptability Evaluation

In study populations that have complex needs, questions of “can it work?” prior to “does it work?” are particularly pertinent [44]. Using mixed methods is beneficial for assessing implementation feasibility, specifically with the inclusion of field notes and observations [45], providing a rich understanding of why the program or methods did or did not work [44].

### Summary

Adapted dance could be a novel adjunct therapy in neurological rehabilitation offering physical and cognitive stimulation and encouraging interactions, in addition to providing other health benefits. However, adapted dance in this field requires strategic program development including co-design techniques. For adapted dance to advance within neurological rehabilitation, essential modifications must occur in consultation with clinicians and patients and reported using standardized methods. Moreover, traditional pre-post research designs should be expanded to include repeated measurements to better understand variability in key outcomes and include safety measures such as fatigue monitoring and management.

### Aims

The aims of this paper are 3-fold: (1) describe a 3-phase co-design approach used to develop the Dance as an Adjunct Therapy for Neurological Rehabilitation – Creative Enrichment for Recovery (DAN-CER) program, (2) provide a description of the resulting DAN-CER program using the TIDieR checklist [35] to promote completeness of reporting for replication, and (3) describe the mixed methods protocol to assess the feasibility and acceptability of the DAN-CER program and the methods used to evaluate its efficacy for improving well-being in addition to monitoring fatigue.

## Methods

### Development of the Program

**Figure 1:**
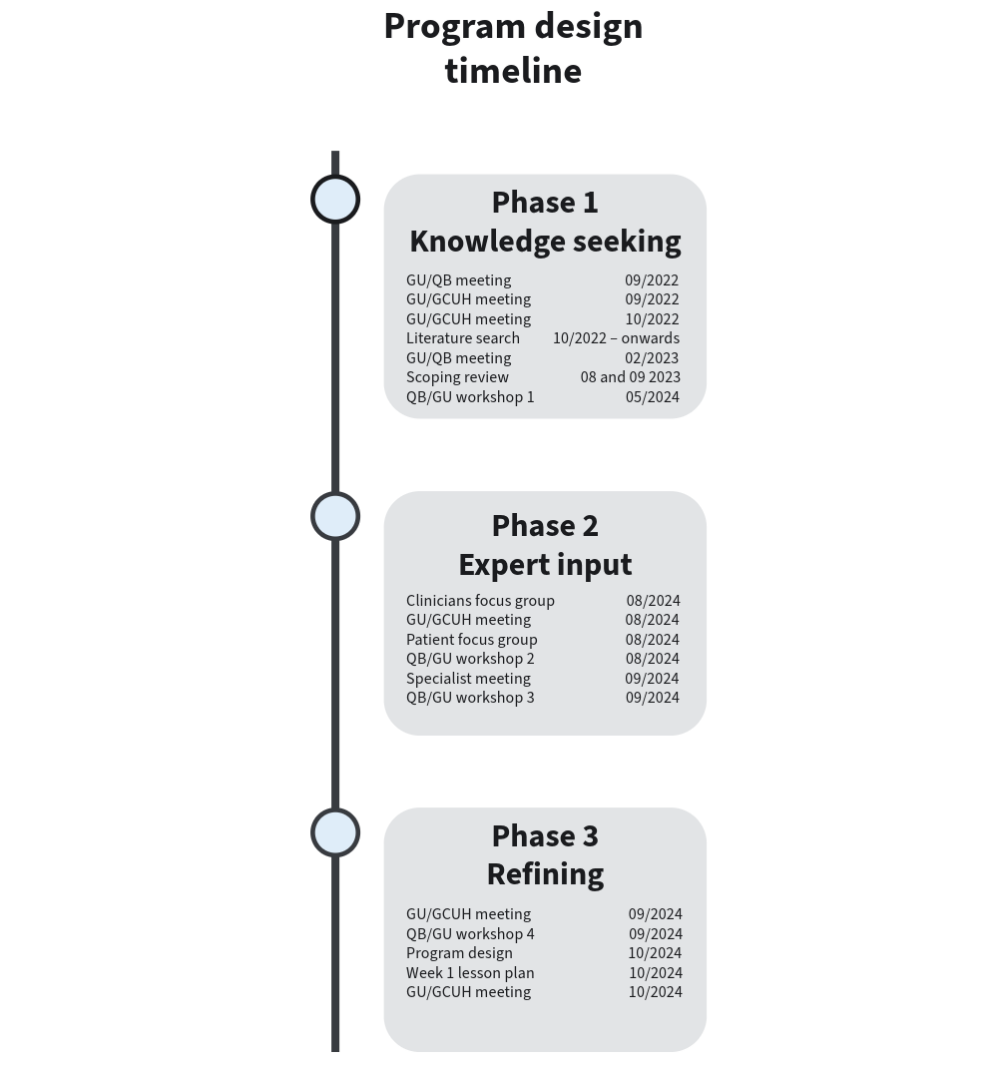
Program design timeline. GU: Griffith University; GCUH: Gold Coast University Hospital; QB: Queensland Ballet.

A 3-phase co-design approach was used to develop the DAN-CER program over 2 years (Aim 1). The phases were (1) knowledge seeking, (2) seeking expert input, and (3) refining. Figure 1 provides an overview of the program development phases.

### Phase 1: Knowledge Seeking

Phase 1 consisted of discussions, a literature review, reflections on previous pilot adapted dance programs, and a teaching artist workshop.

#### Collaborator Discussions

Griffith University (GU), Queensland Ballet (QB), and the Gold Coast University Hospital (GCUH) were involved in initial collaborator discussions to propose formative ideas and assess interest. Discussions occurred during face-to-face meetings in September 2022, October 2022, and February 2023. In attendance were members from the medical and research team (including senior and middle management of the organizations and the GU PhD candidate).

Each party discussed their specific requirements. The university included a PhD candidate who had been involved in the earlier community-based proof-of-concept study—Ballet for Brain Injury (BFBI; a collaboration between Citrine Sun Entertainment, QB, and GU). GCUH required a recreation program that could run on Saturday mornings within their recreation activity program (RAP) using nursing staff to assist. The program needed to be designed for a heterogeneous group of people with acquired brain injuries. The neuroscience rehabilitation ward has a high turnover of patients, so each class needed to be independent from previous and subsequent sessions. It was noted that strict procedures for patients to participate would be necessary. For example, the medical team would need a process to determine who can participate and what movements they could be asked to do. Their procedures required consistent dance teachers throughout the program, with formalized class plans developed ahead of the sessions.

QB preferred a partnership model rather than being contracted to run the program. They proposed including their physiotherapist and expressed concerns about how ballet might be perceived as elite or daunting in a noncreative environment. As a result, they proposed moving away from the term ballet, rather using the more approachable term “dance.”

All collaborators noted resource availability challenges of some nature. Availability of two teaching artists (dance teachers) on a weekend was challenging for QB given their existing weekend program. Similarly, neurosciences ward allied health staffing was restricted on weekends. Funding limitations also restricted the scope and duration of the program.

#### Literature Insights

The literature was searched for useful evidence to support the design of programs for people with neurological impairments. A meta-analysis by Mogan et al [21] concluded that moving in synchrony is beneficial for positive affect and prosocial behavior. A multisensory experience is beneficial following stroke [46], and meditative movement and mindfulness may reduce fatigue in people with brain injury [47]. Regarding movement function, systematic reviews suggest that movement to the beat is beneficial to facilitate motor movements in people with brain injury [48,49] and action observation (observing an action while performing the action) can lead to improvement in movement function [50]. Thus, these concepts became the initial building blocks of the program.

Available dance literature specifically for people with brain injury was reviewed to determine what techniques were currently being used, how dance was being taught, what modifications were used, and if they were effective. This review identified a scoping review on dance with people with stroke [30], but there were no reviews on dance for people with TBI. Therefore, a scoping review was conducted on this topic (D Pretty et al, unpublished data, August 2024). These reviews identified challenges associated with previous neurological dance programs including difficulties initiating movement [34,51,52], risk taking and disinhibition [52], tailoring the intervention (eg, stimulating a stroke-affected side in a heterogeneous class [34]), inappropriate movements during improvisation [34], and the potential that dance increases fatigue [27,29].

#### Pilot Project

A proof-of-concept study, BFBI, was conducted in 2021 (D Pretty et al, unpublished data, December 2024). Follow-up questionnaires were sent to all participants with brain injury and their carers post-BFBI program participation. Responses indicated that people with a brain injury and their carers were satisfied with how the pilot program was delivered. The majority agreed it improved their quality of life and their emotional and mental well-being. Perceived benefits included improved perceived fitness and a sense of social connection, which was linked to positive emotion. Participants reported that the classes were well facilitated and scaffolded. They highlighted the usefulness of movement repetition and the tailored speed of the class to suit the needs of people with brain injury. When asked what could improve the program, one person suggested increased options to suit varying ability levels, and another suggested recapping the previous week due to memory limitations (D Pretty et al, unpublished data, December 2024).

The author DP audited the adapted dance classes throughout the proof-of-concept BFBI study and had informal discussions with the participants and therapists following sessions. These observations highlighted a need to reduce the burden on those assisting participants perform the movements. Specifically, helpers struggled with not knowing what movements were coming next. It was also challenging to maneuver participants (eg, changing sides on the ballet barre). Thus, it was decided that the teaching model required slight modifications to support those assisting dancers and ensure less effort was required.

#### Teaching Artist Workshop

This phase was concluded with a face-to-face workshop in May 2024 with QB teaching artists, research team members including the QB physiotherapist, and the GU researcher/dance teacher (DP). Initial ideas for addressing the program-related challenges were discussed. Optimal approaches for including those with hemiparesis were workshopped. The use of improvisation was also discussed, and consideration was given to how this could be safely implemented.

### Phase 2: Seeking Expert Input

The information gathered from Phase 1 shaped design questions that were further explored in Phase 2 where expert opinion was sought from allied health clinicians (n=2) and neurosciences ward inpatients (n=3) during separate focus groups and a speech pathologist through a meeting. Data from the focus groups were analyzed and applied over 3 steps: (1) Content analysis was used to derive key safety and organizational concerns, including barriers and enablers to patient attendance; (2) conclusions were validated through specialist consultations; and (3) QB/GU workshops were conducted to apply the knowledge gained to program design and implementation.

#### Clinician Input

Two allied health clinicians (a physiotherapist and an occupational therapist) attended a 1.5-hour focus group in mid-August 2024. The clinicians had between 8 years and 19 years of experience in neuroscience rehabilitation, one with and one without previous experience with dance. Example topics in the focus groups were the barriers and enablers to dance, potential benefits of dance, safety concerns, and movements that should be omitted.

Example recommendations made by therapists were that movements be gentle and stay within their base of support (no leaning forwards or sideways) and that exercises avoid the need for arms or legs moving at the same time (eg, feet marching without arms or arms extended without feet marching). The need for modifications to language such as slowed pace of instruction, the additional use of gestures, and using language sensitive to those who have had a limb amputated were suggested. Views on the appropriateness or inappropriateness of patients performing movements standing if able were raised, and a safety modification of no passive assistance for those with hemiparesis was recommended.

Concerns were raised regarding dance teachers’ lack of medical knowledge or medical training and how this might impact patients. For example, if teachers would instruct patients to “stretch” when this would be contraindicated for them or if they would give unsolicited advice regarding what would be beneficial for patients from a rehabilitation perspective. The onsite location of the dance classes was also discussed. For example, concerns included the impact of using the therapy room on a weekend; logistical challenges such as transporting the patients to and from the location; and equipment safety and staffing limitations on the weekend. These important topics raised were then discussed with the medical team, and a plan of action for each topic raised was devised. This involved medical emergency team call procedures and processes for the research nurse to provide dance teachers with information regarding what movements patients are and are not cleared to do or any specific necessary precautions.

#### Neurosciences Inpatient Input

A 60-minute focus group was attended by 3 neurosciences ward inpatients with acquired brain injuries (56-77 years old; 2 women, 1 man) in mid-August 2024. Questions pertained to what they wanted to achieve, what would help them feel safe, what type of dance movements and music they wanted, and what barriers there would be to participating. Patients were given the questions earlier in the day to allow time to plan their answers. Issues raised included the desire for simple movements (such as percussion) for inclusivity, repetition, and improvisation. They discussed that having themes would be fun and something to look forward to with certainty. When discussing music, the patients reminisced about music, dance halls, and music from their younger years. It became apparent that music selection from relevant eras could be an important element to the adapted dance classes.

Three other salient topics patients raised included agency, boredom, and safety. Patients discussed that, during inpatient care, every element of their day is chosen for them, from what time they shower to what time they eat. The opportunity to choose and have influence over decisions such as the opportunity to create movements or choose music was important. The desire to break the boredom of “staring at the ceiling” was also relished. Regarding safety, patients discussed the desire for clinicians to be involved in their lessons. They also described potential barriers of fatigue, pain, and sickness.

#### Specialist Support

Based on clinician opinions regarding the need to tailor communication for people with brain injury, specialist support was sought from a speech pathologist at GCUH through a 60-minute virtual meeting in early September 2024. Suggestions included the use of both verbal cues and gestures to communicate, using repeated simple instructions on repeated movements (eg, arms, arms, arms), and the use of concrete descriptions free of jargon. Suggestions to support attentional processes included having only one main speaker for each section, using a consistent technique to get attention, and keeping distractions to a minimum.

Advice for supporting cognitive disorders included segmenting teaching, where one teaches the more complex version and one teaches a simpler version instead of demonstrating both options and allowing the patient to choose. Given the potential challenges associated with the use of improvisation and movement creation, it was suggested that a contingency plan be developed for the improvisation section including alternative facilitation strategies.

#### Teaching Artist Workshop

Two workshops were conducted with QB teaching artists, a QB physiotherapist, and the GU researcher/dance teacher (DP) in August 2024 and October 2024 to consolidate all information learned. Together, this information was used to develop the *what* and *how* of the program and classes. Teaching techniques were refined and practiced at these workshops. The structure of the class was determined, and fundamental movements to be included in the movement bank were decided.

### Phase 3: Refining

In Phase 3, the aim was to build on and finalize the adapted dance program and the movement bank and to manualize the program (the TIDieR checklist and class plans). The weekly sessions could then be built to these parameters with confidence in safety and consistency in sessions. To enact this, initial concepts were disseminated (via movement and teaching videos and the TIDieR checklist). Feedback was then gathered and implemented in an iterative process, alternating between meetings and workshops. In collaborative meetings (which included further expert support from allied health and a rehabilitation team member), processes for safe participation were further developed where necessary.

#### Movement Bank

The safety of the preliminary devised movements was assessed through feedback from the medical research team. A video bank, consisting of prototypical movements from each section of the class (ie, mindful warmup, percussion, arms and legs) was shared, along with the corresponding cueing styles. The videos included a range of potential movements of differing degrees of challenge, by difficulty (levels 1, 2, and 3).

#### GCUH Medical Team Feedback

In late September 2024, feedback on the movement bank was given through an online meeting with members of the medical research team who had also consulted other specialist rehabilitation team members. This feedback was predominately on providing additional modifications for those in wheelchairs and for those with upper limb mobility challenges. Other modifications included the use of headset microphones for the hearing impaired and the requirement of additional stability aids if some patients were to be standing. The expected proportion of patients who would be able to participate in certain exercises or not was also discussed for dance teacher knowledge.

#### Teaching Artist Workshop

Final program design based on this feedback occurred in a 2-hour face-to-face workshop in late September 2024. Further cues for the creative section were mapped out, as were the themes and design of the repertoire section. Music options were discussed, and a music bank was initiated for quick selection over the weeks in case any in vivo adaptations needed to be made to the song tempo. Following this, the TIDieR [35] was completed, and preliminary weekly class plans (which included cueing and any precautions) were devised collaboratively online between the QB and GU team members then sent to the hospital team for final approval.

#### GCUH Medical Team Feedback (2)

Further feedback on the program was given (such as the removal of neck movements (eg, slow looking sideways). Standing exercises were removed for the first week of the program.

### Outcomes

At the end of each phase, critical decisions about the program were made. Table S1 Multimedia Appendix 1 reports these decisions. The need for additional processes was also highlighted and devised. For example, to assist with patient eligibility as to who can perform what level of movements, it was agreed that the class plan for the week would be sent to the medical research team on Monday afternoons ready for their Tuesday morning staff discussions. Table S2 in Multimedia Appendix 1 highlights the safety concerns between stakeholders and how these were addressed.

### The DAN-CER Program Mapped to the TIDieR Checklist

Following the end of Phase 3, the DAN-CER protocol was finalized. In this section, a description of the DAN-CER program was mapped to the TIDieR checklist to promote completeness of reporting for the purposes of replication (Aim 2). The protocol is described in Table S3 in Multimedia Appendix 1. Using the TIDieR checklist ensured that clinicians, allied health professionals, and dance teachers can replicate or build on research findings. 

### Protocol for Piloting the DAN-CER Program

This section outlines the protocol to pilot the DAN-CER program (Aim 3). The primary objective is to assess the feasibility and acceptability of DAN-CER in the subacute hospital setting using a mixed methodology. The secondary objective is to pilot a single-case, 1-phase (B phase), nonexperimental case methodology to assess the efficacy of DAN-CER for improving well-being in addition to monitoring fatigue.

#### Registration

The proposed pilot is not a clinical trial and, therefore, has not been registered. Any future evaluative research on the DAN-CER program will be registered.

#### The Brain and Enriched Environment Laboratory

This project is a Brain and Enriched Environment Laboratory (BEEHive) project. As described by Norwood et al [11], the BEEHive is a multidisciplinary team situated within The Hopkins Centre, GU, that investigates how an enriched environment can assist neurological rehabilitation. Specifically, the BEEHive aims to develop (using a participatory research approach), test (including piloting and acceptability and efficacy testing), and deliver novel solutions to enrich the neurorehabilitation environment.

#### Setting

DAN-CER operates at the GCUH neuroscience rehabilitation unit as part of their RAP. This is a 28-bed unit in which the hospital team provides intensive inpatient rehabilitation throughout weekdays and recreational activities occur on weekends. The classes are held on the neuroscience ward on Saturday mornings at 10 AM for 6 weeks and began in mid-October 2024.

#### Study Design

The study uses a mixed methods design. Mixed methods were chosen to generate comprehensive insights that may be used to inform further adaptations of the DAN-CER intervention and methodology. The mixed methods design includes semistructured interview questions, field notes, and quantitative data (eg, questionnaires and count data). Regarding quantitative data, the study incorporates a single-case, 1-phase (B phase), nonexperimental design with replication across 3 participants. Interparticipant replication is chosen for intensive study at the individual level to capture different characteristics of brain injury. This approach provides a detailed understanding of any potential impact of the program while also assessing how variable outcomes are from day to day and differences between participants. The chosen methodology (as opposed to an A-B or multiple baselines study) is due to a range of contextual factors such as the average patient length of stay, the available time for running the classes, and funding. In addition, restricting enrichment in a group of people that have minimal other activity options (due to delivery of the adapted dance program occurring on the weekend when no therapy is available) could raise ethical concerns in terms of potential negative emotional impacts [53], which we aimed to avoid. Given the pilot nature of the case study methodology, there is no control group and therefore no randomization nor blinding. Results will give an indication of how to proceed with future iterations of the study.

**Figure 2:**
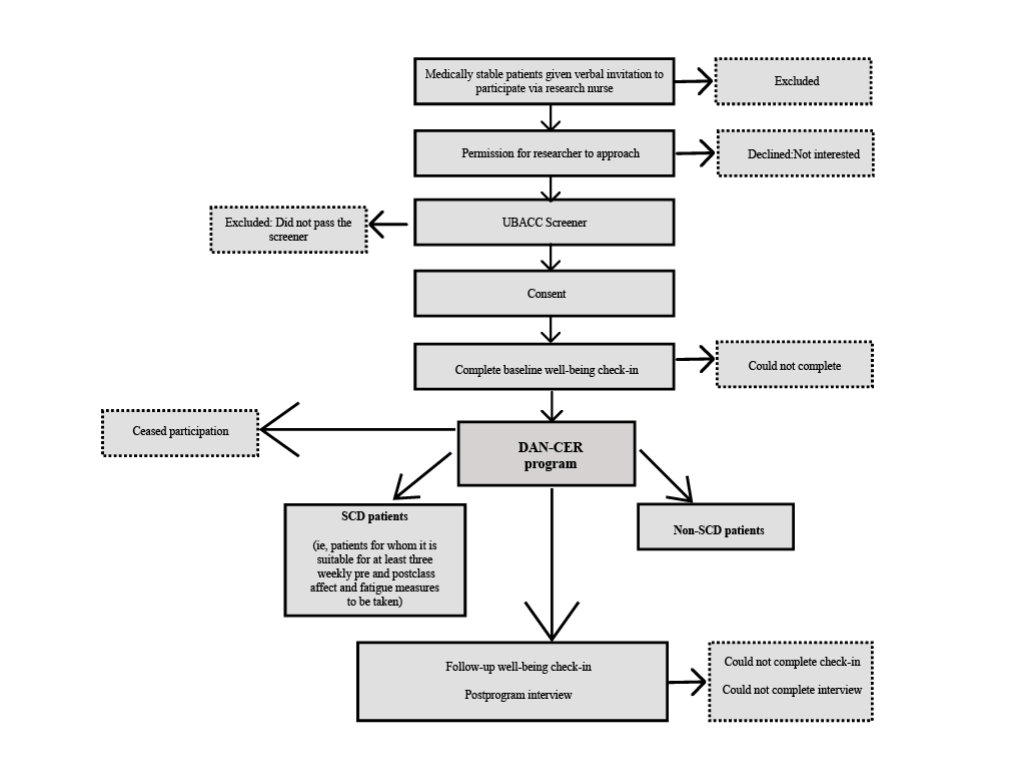
Flowchart of the protocol for neuroscience ward patient participants. DAN-CER: Dance as an Adjunct therapy for Neurological rehabilitation – Creative Enrichment for Recovery; SCD: single case design; UBACC: University of San Diego, Brief Assessment of Capacity to Consent.

By using a mixed methods pilot, any issues with data collection (eg, due to logistical or communication barriers, time pressures, fatigue) and program implementation can be evaluated and used to inform the design of future research methodology, including outcome measures (for example, determining if a single-case experimental design is feasible) and any further program modifications needed. See Figure 2 for the schematic diagram of the study process and Table 1 for the schedule of assessments.

**Table 1:** Schedule of enrollment, assessments, and adapted dance classes.

Activities	Time (minutes)	B1^a^	Week 1 class		Week 2 class		Week 3 class		Week 4 class		Week 5 class		Week 6 class		F1^b^
			A^c^	B^d^	A	B	A	B	A	B	A	B	A	B	
Consent	30	✓	—^e^	—	—	—	—	—	—	—	—	—	—	—	—
PANAS^f^	5	—	O^g^	O	O	O	O	O	O	O	O	O	O	O	—
VAS-F ^h^	5	—	O	O	O	O	O	O	O	O	O	O	O	O	—
DASS-21^i^	5	✓	—	—	—	—	—	—	—	—	—	—	—	—	✓
WEMWBS^j^	5	✓	—	—	—	—	—	—	—	—	—	—	—	—	✓
Exit interview	<30	—	—	—	—	—	—	—	—	—	—	—	—	—	✓

^a^B1: pre-Dance as an Adjunct therapy for Neurological rehabilitation – Creative Enrichment for Recovery (DAN-CER).

^b^F1: post-DAN-CER follow-up

^c^A: pre-adapted dance class assessment.

^d^B: postadapted dance class assessment.

^e^Not applicable.

^f^PANAS: Positive and Negative Affect Scale.

^g^O: option to assess at this time if appropriate (eg, some patients may not be assessed every week).

^h^VAS-F: visual analog scale for fatigue.

^i^DASS 21: Depression Anxiety Stress Scales.

^j^WEMWBS: Warwick-Edinburgh Mental Wellbeing Scale.

#### Participants and Recruitment

The study includes 2 groups of participants, as follows: (1) patients at the GCUH who sustained a brain injury and are residing on the neurosciences ward and (2) neurosciences ward staff who assist with program delivery. The group of patients will primarily consist of people who have experienced a stroke, TBI, or neoplasm. A research nurse provides information about the project to the patients. As a pilot study, the adapted dance classes are adjunct to participants’ other therapies. For the staff group, any staff member present during the adapted dance sessions receives information about the follow-up interview at the end of the day. All participants are adults (>18 years) who can communicate in English.

Patients residing on the neurosciences ward are considered a special population necessitating additional ethical considerations, as discussed in the following paragraphs.

Regarding cognitive impairments, to be eligible, patient participants must demonstrate sufficient cognitive capacity to understand the study’s purpose and provide informed consent. Eligibility requires completion of the University of California, San Diego Brief Assessment of Capacity to Consent (UBACC) [54], with a passing score. Further, a patient consent form modified with assistance from a speech therapist is used to ensure that the language used is appropriate.

For recruitment considering the dependent relationship with hospital staff, eligible patients are approached by a research nurse, who obtains verbal consent to allow a university researcher to provide further information.

Patient data are maintained in accordance with an approved data management plan to ensure confidentiality and security. When required, essential data are securely transferred to co-investigators via password-protected links, granting access to the individual research files stored in a secure system.

#### Sample Size

The sample size required for the study is not derived statistically but rather determined by feasibility and pragmatic considerations. The sample size is constrained by class size parameters for safety (a maximum of 6 participants per session) and an expected high turnover of patients during the 6-week period (due to discharge from the ward). Accrual data are gathered from all patients on the ward, and qualitative data are gathered from up to 10 patients and nursing staff who participated in the program.

#### Intervention and Procedures

The intervention is outlined in Table S3 in Multimedia Appendix 1 in accordance with the TIDieR checklist [35]. Several potential challenges to implementation are anticipated and addressed. The most likely concern is staff shortages due to sickness. In such instances, a contingency plan is in place to reduce the number of participants in each class, ensuring that the appropriate staff-to-patient ratio is maintained. Additionally, if logistical issues such as transportation difficulties arise, the class is relocated to the dining room, which is closer to patient rooms.

Another key consideration involves the burden on clinical staff to conduct patient clearances. This challenge is minimized by aligning the clearance process with the existing procedure for granting weekend leave. To facilitate this, the lead author developed a proforma document for completion.

#### Outcomes

To align the research questions with the quantitative and qualitative data sources, a data source table has been developed. This is presented in Table S4 in Multimedia Appendix 1. Further descriptions are provided in the following sections where necessary.

#### Primary Objective Measures

##### Feasibility

Feasibility is gauged through recruitment, retention, and attendance. Here, field notes provide a deeper understanding. For example, if a participant ceased participation during a class, the reason is noted (eg, fatigue, nonenjoyment, conflicting schedule). These data on attendance and field notes are collected by the researcher/dance teacher (DP). Regarding accrual success criteria, ongoing feasibility for the RAP requires at least 6 participants who are deemed safe and interested per week. To justify staffing, a minimum of 4 patients per class is needed. These numbers serve as the feasibility targets. Feasibility assessment also includes any safety concerns such as adverse events or minor harms.

##### Acceptability

Acceptability is assessed through qualitative analysis of face-to-face interviews with patient participants (target numbers of 6 individuals with acquired brain injury) and neurosciences ward staff (target number of 12). All patients with brain injury who participate in the adapted dance sessions are given the opportunity to provide insight into their experience with the program through a semistructured exit interview. Similarly, all staff involved in the program delivery are invited to provide feedback. Example topics for questions to participants include their overall experiences, likes and dislikes, any challenges and how these were overcome, and whether they perceive any benefits. Example topics for questions to staff include their general observations and experiences; perceived benefits or disadvantages to the participants, themselves, or the operation of the hospital; and any other feedback. Acceptability is also assessed through dance teacher, research nurse, and multidisciplinary team notes.

#### Secondary Objective Measures

##### Psychosocial Outcomes and Fatigue

Participants who can complete the repeated assessments have fatigue and affect assessed before and after each adapted dance class for the duration of their participation. It is expected that approximately 3 sets of complete pre and postsession individual data will be gathered across the 6-week program. Fatigue is assessed using the visual analog scale for fatigue (VAS-F) [55], and affect is assessed using the Positive and Negative Affect Scale (PANAS) [56].

The VAS-F will be used to assess present moment fatigue. On it, participants circle their response (0 to 10) to 18 items indicating how energized and fatigued they feel. Responses range from “not at all” to “extremely.” The VAS-F has been found to have convergent validity with the Standford Sleepiness Scale and the Profile of Mood States and has high internal consistency. Cronbach α for a patient group was 0.96 and 0.94 for evening and morning assessments, respectively. Results can be interpreted according to two subscales, an energy subscale and a fatigue subscale. Where necessary, the researcher reads aloud the questions and circles responses on their behalf.

The PANAS [56] asks 20 questions about current mood. It measures 11 specific aspects of mood including enthusiasm, pride, fear, and sadness. Participants indicate the extent that they feel a certain emotion at that present moment. Responders choose from “very slightly or not at all,” “a little,” “moderately,” “quite a lot,” or “extremely” (1 to 5, respectively). Positive and negative affect items are then summed, producing a score between 10 and 50. The present moment internal consistency is 0.89 for positive affect and 0.85 for negative affect. The PANAS has been used to measure daily affect changes in individuals with brain injury [40].

All neuroscience ward patient participants recruited for the adapted dance intervention undergo mood and well-being assessments assessed at baseline as a “wellness check-in” using two validated measures. These scales are the Depression Anxiety Stress Scales (DASS-21) [57] and Warwick-Edinburgh Mental Wellbeing Scale (WEMWBS) [58]. Participants are re-assessed if they complete at least 4 of the 6 adapted dance sessions. Completion of both pre and postdata is expected for up to 10 participants.

The DASS [57] asks questions regarding levels of agitation; low mood; apathy; and body sensations related to depression, anxiety, and stress such as dryness of mouth and trembling. Participants respond by circling a response (0 to 3), with 0 representing “did not apply to me at all” and 3 representing “applied to me very much or most of the time.” Due to the population, the shorter questionnaire (DASS-21) will be used, as it takes one-half the time but retains validity. The DASS has been validated in a TBI sample [59], with depression and stress subscales showing adequate internal reliability. The test-retest reliability (taken between 1 week and 3 weeks apart) of the depression, anxiety, and stress subscales in a brain tumor sample was 0.77, 0.65, and 0.60, respectively [59]. The tool’s validity as a screening measure has also been assessed in a TBI population [60].

The WEMWBS [58] has 14 statements regarding positive feelings and thoughts such as optimism, usefulness, self-efficacy, and feeling loved. Respondents reflect on their experience over the last 2 weeks and respond by circling a response from 1 through 5, with 1 representing that they felt that feeling “none of the time” and 5 indicating “all of the time.” It has shown sensitivity to change from hospital-based remediation programs with patients with schizophrenia [61] and has been validated as being responsive at the individual level [62]. Test-retest reliability is 0.83 in the general population [58] and 0.84 in older adults [63]. It has been used with participants with stroke [64].

#### Data Analysis

Approaches to analysis will differ depending on the data type. Pre- and postsession PANAS and VAS-F will simply be plotted and visually displayed. For the pre-and postprogram well-being data, a reliable change index will be calculated (change score divided by the standard error of the two measurement scores) [65]. Recruitment and attendance will be displayed in a flowchart, showing the total number admitted to the ward during the study period, the number deemed eligible and offered the program, the number consenting to participate, and those who attended. The average number of classes attended over the 6 weeks will also be presented.

Interviews will be transcribed verbatim and deidentified for analysis. Interview transcripts will be analyzed thematically using the method proposed by Braun and Clarke [66] to devise themes concerning participants’ perceptions of the adapted dance program. DP and MN will initially read and reread interview transcripts to immerse themselves in the data. Initial codes, along with example raw data, will then be presented in a team meeting for team level discussion and code refinement. Themes will be collaboratively developed by the research team.

#### Mixed Methods Data Integration

As recommended by Aschbrenner et al [44], a joint display table will be produced where rows represent each aspect of feasibility (ie, accrual, retention, attendance, safety, and acceptability) as well as pilot efficacy and measurement and design feasibility. Columns will represent quantitative data (including count data and reliable change index), qualitative data (stratified via stakeholder) including dance teacher and research nurse notes, and themes raised in the semistructured interviews. The final column will be the point of intersection or integration of quantitative and qualitative data including a comment on where the data diverge or are in agreement.

In the case study write-up, qualitative data will be used to expand on the quantitative data in a side-by-side write-up where the patient-specific qualitative data can add value to the quantitative data.

### Ethical Considerations

This study has ethics approval (HREC/2023/QGC/99631 and GU 2023/813). All participants provide written consent and can withdraw at any stage. For their participation, participants are offered entry into a draw to win 1 of 3 gift cards worth 50 Australian dollars (~32 USD).

Files are stored in a secure system, and when required, essential data are securely transferred to co-investigators via password-protected links.

### Dissemination

The dissemination plan uses multiple strategies to ensure widespread access of the findings among key stakeholders. All participants will be given the opportunity to receive a summary of the results. In addition to traditional dissemination methods such as peer-reviewed journal publication and conference presentations, the results will also be shared with hospital staff internally through the BEEHive laboratory newsletter, online presentations with senior staff, and at The Hopkins Centre events hosting relevant brain injury associations.

### Reporting

The SCRIBE (Single Case Reporting Guideline In Behavioural Interventions) reporting guideline was not intended for a nonexperimental, single-case study design; however, as highlighted by Tate et al [67], following the guidance of the items may be useful. Specifically in this case, where assessing the feasibility of a future single-case experimental design study is an aim, reporting relevant items according to the guideline is advantageous. Therefore, where applicable, the proposed study will be reported in line with the SCRIBE guidelines.

Patient demographic characteristics will be reported in detail including clinical characteristics such as the cause or type of acquired brain injury; baseline participant characteristics such as functional status, age, and gender; and baseline DASS and WEMWBS scores.

## Results

Data collection for the pilot began on October 21, 2024. The classes ran from October 26, 2024, to December 6, 2024. During this period, 9 patients participated in at least one adapted dance class. Data collection is now complete. Follow-up interviews were conducted with 8 patients and 5 nurses. All interviews have been transcribed, and preliminary coding is now underway, with initial codes being tentatively developed and refined through immersion in the data. The results of the study are expected to be submitted for publication in December 2025. This research received no external funding.

## Discussion

### Overview

Dance combines physical, cognitive, and social activity, and for many, dance is considered an enjoyable, fun experience, thus making it a potential novel environmental enrichment activity for those in inpatient neurological rehabilitation. However, adapted dance program design, feasibility and acceptability assessment, and efficacy assessment in the neurorehabilitation setting need to be systematic and involve end users throughout development. Thus, there were 3 aims in this paper that address these issues.

Aim 1 was to report the design and development of the DAN-CER program. In Aim 2, the resultant program was mapped to the TIDieR checklist [35] for transparency. The information consolidated from the stakeholders across the 3 phases shaped the design of the DAN-CER program. Each stakeholder contributed uniquely. Input from allied health staff assisted designers with developing strategies to suit participants’ learning capabilities (eg, the use of short sequences, hand gestures, attention aids). Input from clinicians assisted with improving patient safety (eg, movement omissions and suggestions) and fine-tuning the operational processes. Input from current patients ensured that future neuroscience ward patients would find the program interesting and manageable (through the use of themes, free choice in music selection, and movement style). By using this inclusive approach, it is hoped that the final program will accommodate the physical, cognitive, and communication challenges patients may face.

Aim 3a related to developing protocols for assessing how appropriate the adapted dance program is in the neurological inpatient setting (primary findings). Teresi et al [45] described that, in diverse populations, it is imperative to assess the feasibility and acceptability of an intervention before scaling up. This is a step that is often missed in adapted dance literature, although see [27,29,34]. Continuing the conscious decision to include all stakeholders’ viewpoints, the study protocol is a mixed methods design whereby all perspectives are integrated. Accordingly, insight into what changes need to be made to the classes (or the processes to carry out the classes) is captured from the full range of people that the classes will impact, thus providing a comprehensive indication of whether and how future studies should proceed.

Aim 3b reports the protocol for piloting a single-case, 1-phase (B phase), nonexperimental design methodology to assess the efficacy of DAN-CER for improving well-being (using the WEMWBS and PANAS) in addition to monitoring fatigue. These secondary findings will be used to design future rigorous efficacy studies. To date, no known adapted dance study has investigated positive psychological constructs quantitatively or used a single-case methodology that includes repetitive testing to capture changes in well-being in the context of an adapted dance program and how variable these impacts are in a brain-injured population. Based on the cohort, it is expected that our pilot data will reflect high variability (inter and intraperson), and, for this reason, a single-case methodology will be a suitable approach in future studies. The primary outcomes (feasibility) will assist with the decision-making process regarding whether there is enough interest, consistent attendance and treatment, and minimal to no adverse effects for a more rigorous single case experimental study and what type of design would be appropriate.

### Strengths and Limitations

The use of mixed methods is the main strength of the pilot study. This methodology allows for the integration of data sources to highlight where the feasibility and acceptability results diverge or converge stratified by stakeholder and within stakeholder but across different data types (qualitative and quantitative). The main limitation of both the program development and the proposed protocol study is that it uses convenience sampling, consisting of a small sample size. Further, the research protocol is not experimental in the sense that it does not involve randomization, a control arm, or a baseline period. Therefore, interpretation of outcomes will require caution.

### Future Studies

In the current DAN-CER program, we consciously designed an adapted dance program that uses simple synchronous movement as its main pillar, in the hope of creating an environmental enrichment activity for the inpatient neurological population to benefit mood and well-being. Beyond further iterations including more rigorous efficacy studies, different aspects of dance (eg, cognition, movement, social interaction, rhythm) can be strategically manipulated to assess the impact on neuroplasticity in the damaged brain. Further, questions around the optimal cognitive and physical load in the adapted dance program and timing in the rehabilitation pathway represent valuable future research directions. Before such questions can begin to be answered, there is a need for stakeholder-inclusive design, transparent reporting of program design, and rigorous methodology that assesses outcomes at the individual level.

### Conclusion

This paper represents the first step of many, whereby an adapted dance program for use in inpatient neurological rehabilitation is ethically designed, transparently reported, and tested for acceptability. Future studies will assess its efficacy more rigorously.

## Appendix

Multimedia Appendix 1 Decisions, resolutions, program details, and data sources.

## Abbreviations

BEEHive: Brain and Enriched Environment Laboratory
BFBI: Ballet for Brain Injury
DAN-CER: Dance as an Adjunct therapy for Neurological rehabilitation – Creative Enrichment for Recovery
DASS-21: Depression Anxiety Stress Scales
GCUH: Gold Coast University Hospital
GU: Griffith University
PANAS: Positive and Negative Affect Scale
QB: Queensland Ballet
SCRIBE: Single Case Reporting Guideline In Behavioural Interventions
TBI: traumatic brain injury
TIDieR: Template for Intervention Description and Replication
UBACC: University of California, San Diego Brief Assessment of Capacity to Consent
VAS-F: visual analog scale for fatigue
WEMWBS: Warwick-Edinburgh Mental Wellbeing Scale

## Data Availability

Anonymized transcripts of focus groups are available on request. Any identifying comments such as role will be removed.
