# Supplementary material for: Dance as an Adjunct Therapy for Neurological Rehabilitation – Creative Enrichment for Recovery (DAN-CER): Program Design and Protocol for a Mixed Methods Pilot to Assess Feasibility and Acceptability
Source: JMIR Res Protoc. 2025 Aug 26;14:e69452. doi: 10.2196/69452 (PMC12421208; doi:10.2196/69452)
Supplement: Multimedia Appendix 1 [file resprot_v14i1e69452_app1.docx]

# Multimedia appendix

**Table S1.** Summary of program decisions based on stakeholder feedback.

|  | Decisions | GU | GCUH  - A | NSWP | GCUH  - B | QB |
| --- | --- | --- | --- | --- | --- | --- |
|  |  |  |  |  |  |  |
| Cognitive |  |  |  |  |  |  |
|  | Give cues to staff first, then patients |  | ✓ |  |  |  |
|  | No expectation of participants to remember sequences; movements will always be cued | ✓ |  |  |  |  |
|  | Only one teacher speaks at a time |  | ✓ |  |  |  |
|  | Plan short sequences | ✓ | ✓ |  |  |  |
|  | Reduce distractions and monitor noise |  | ✓ |  |  |  |
|  | Use a consistent clapping sequence to gain attention |  | ✓ |  |  |  |
|  | Use instrumental music for better concentration when needed | ✓ | ✓ |  |  |  |
|  | Use music to direct attention (e.g., note a movement on the chorus or upcoming crescendo) | ✓ |  |  |  |  |
|  | Write the class plan up on board in large font |  | ✓ |  |  |  |
| Communication |  |  |  |  |  |  |
|  | One teacher demonstrates one level, one demonstrates the other options to reduce confusion |  | ✓ |  |  | ✓ |
|  | Use common hand gestures to accompany verbal cues |  | ✓ |  |  |  |
|  | Repeated cuing | ✓ | ✓ |  |  |  |
|  | Use concrete descriptors | ✓ | ✓ |  |  |  |
|  | Use slowed, simplified language |  | ✓ |  |  |  |
| Conceptual |  |  |  |  |  |  |
|  | Multisensory stimulation | ✓ |  |  |  | ✓ |
|  | Promote agency by offering choices in music and movement |  |  | ✓ |  | ✓ |
|  | Promote social connection and positive mood by moving in time with others | ✓ |  |  |  |  |
|  | Promote social connection through shared focus and collaboration | ✓ |  |  |  |  |
|  | Use themes each week |  |  | ✓ |  | ✓ |
| Design |  |  |  |  |  |  |
|  | Offer varying levels of complexity | ✓ | ✓ |  | ✓ | ✓ |
|  | Design for a mixed ability class | ✓ |  |  | ✓ | ✓ |
| Movements |  |  |  |  |  |  |
|  | Include mindful movement sections to reduce fatigue | ✓ |  |  |  | ✓ |
|  | Include mirroring exercises | ✓ |  |  |  | ✓ |
|  | Limit movements to within their base of support |  | ✓ |  |  |  |
|  | Minimize head and neck movements |  |  |  | ✓ |  |
|  | Move arms or legs, not both simultaneously |  | ✓ |  |  |  |
|  | Use gentle movements | ✓ |  | ✓ |  |  |
|  | Use repetitive movements | ✓ | ✓ | ✓ |  |  |
|  | Use simple movements |  | ✓ | ✓ |  |  |
| Music |  |  |  |  |  |  |
|  | Use music with a strong beat | ✓ |  | ✓ |  |  |

Note: GU = Research work from Griffith University; GCUH-A = Input from allied health (derived from the focus group) and specialist support meeting; GCUH-B = Feedback from the GCUH clinical and multidisciplinary team; NSWP = Input from neurosciences ward patients (derived from the focus group); QB = Queensland ballet teachers and physiotherapists.

**Table S2.** Examples of challenges and resolutions in the program design and implementation processes.

|  | Challenge/concern | Response/Resolutions |
| --- | --- | --- |
|  |  |  |
| **Cross discipline collaboration** |  |  |
|  | Concerns were raised regarding the more limited medical knowledge of dance teachers. | Medical oversight will be provided throughout the program’s design and implementation. For example:  a. Safe movement selection: A video bank of exemplar movements, categorized by complexity, was created for the medical team to review and provide feedback. The program was also manualized (mapped to the TIDieR checklist)[1], allowing the medical and rehabilitation team to provide feedback prior to program onset.  b. Patient eligibility: The medical team will determine participant suitability for each week’s class.  c. Monitoring: Clinical nursing staff will attend all classes, being aware of each patient’s current level of function and any restrictions identified by the allied health team. They will monitor and collaborate with teachers as needed. |
|  | Concerns were raised about the professionalism of the dance teachers including the potential for offering unsolicited medical advice to patients. | a. Teaching Artists of Queensland Ballet are not permitted to give unsolicited medical advice, as per their position descriptions and company Code of Conduct. Teaching Artists will additionally undergo an induction outlining clear roles, including the expectation that no medical advice will be given to patients.  b. Clinical nursing staff will be present for the duration of each class, monitoring and collaborating with teachers and patients as needed.  c. To ensure consistency and professionalism, the project will employ a small team of highly experienced dance teachers. |
| **Weekend operations** |  |  |
|  | It is likely that the allied health staff will be unavailable on the weekends. Safety concerns were raised especially regarding fall risk particularly with standing exercises. | In response to concerns, it was decided that the program can run safely without allied health present during the classes. The following measures will be implemented:  a. Rehabilitation nursing staff will be present for all classes on a 1:4 ratio, as well as the Recreation Activity Program assistant in nursing to support teachers and patients.  b. In the first week of the intervention, all patients will remain seated regardless of mobility. A post-session meeting with the medical and allied health team will then assess initial concerns and evaluate the feasibility of incorporating standing exercises. |
| **High turnover of patients** |  |  |
|  | New patients will join each week, alongside continuing patients. | A process will be used to safely manage the rolling start of each patient and ensure that each week’s class plan meets the individual needs of that cohort. For example:  a. Dance teachers will send their class plan to clinical and allied health staff by Monday afternoon for the Saturday class.  b. During the week, clinicians will select suitable patients and highlight any contraindications. Dance teachers will be informed of vital details, such as which patients have impulsive mobility, so that these patients can be seated with nurses positioned between them. |
|  | Dance teachers will need to manage the diversity in patient conditions from week to week. | Dance teachers will plan ahead to be adaptable. They will include pre-designed modifications in weekly class plans and devise a music bank with multiple tempo options for each exercise to make modifications easier. |
|  | The dance classes will need to be inclusive to those who start the program late. | Each session will follow a consistent structure but remain self-contained, with no reference to or progression from previous sessions. Each week will introduce entirely new exercises ensuring that participants can fully engage without requiring prior knowledge or skills from earlier sessions. |
| **Mixed ability class and Resource limitations** |  |  |
|  | In the focus group, concerns were raised regarding the potential for passive assistance of patients during movement. | It was decided that neither the nursing staff nor dance teachers would provide passive assistance, but the program would still remain inclusive of individuals with limb weakness. This will be achieved through self-multimodal stimulation (tapping of a limb in time to the music). |
|  | The burden on the assistants helping the patients will need to be minimal. | The program will minimize the burden on nurses supporting patient participation by:  a. Reducing position changes, such as moving between areas of the room.  b. Pre demonstrating and cueing each exercise to ensure RAP nurses, clinical nurses, and patients know what to expect.  c. Limiting standing exercises (if used) to one stand by assist patient per exercise. |
| **Location** |  |  |
|  | The therapy room is the preferred location, however transporting patients there presents operational challenges. Dance teachers are not trained to operate wheelchairs, and nurses cannot leave patients in the care of teachers while transporting others. | To use the therapy room, assistance will be required for patient transportation before, after, and during the class (if they need to leave mid-session). Therefore:  Wards persons on the unit will be designated to assist with transportation of patients before and after the classes and can also be called throughout to assist - ensuring clinical staff remain available for the class. |
|  | The concern was raised that if nurses leave the ward to assist with the dance class in the therapy room, it may result in understaffing on the ward. | The management team reviewed this concern and determined that, with the current nurses to patient’s ratio, and the assistance of an additional clinical nurse in charge, staffing will remain sufficient during the class, with no increased risk. |
|  | A concern was raised regarding how to manage a medical emergency in the therapy room. | A multidisciplinary team determined that clinical nurses, fully competent and knowledgeable of emergency protocols, will be present during the dance class to manage any medical emergencies in accordance with health and human services emergency protocols.  Specifically, the gymnasium space has a policy for MET calls which addresses off ward emergency events. |
|  | There were differing opinions on the appropriateness of dance teachers using the therapy room without allied health staff present. | A multidisciplinary team determined the following:  a. An induction will be conducted to outline which areas of the therapy room can be used and clarify that no equipment should be touched.  b. Clinical nursing staff will be present to monitor use and ensure the area is cleaned before vacating. |
| **Fatigue** |  |  |
|  | Fatigue is a known barrier and safety concern for patients in neurological rehabilitation. | Program development will incorporate strategies to minimise fatigue:  1.Because of the link between cognitive load and fatigue, the program will minimise cognitive demands, such as;  a. Providing both verbal and physical cues.  b. Limiting movement sequences to a few movements to avoid memory overload.  c. Modifying exercises to reduce dual tasking if necessary.  2. Mindful movement will serve as the foundation of the program to help reduce fatigue. |
|  |  | A multifaceted approach to fatigue management will be implemented in the dance class.  a. If a patient is fatigued prior to class, their participation will be reassessed.  b. A rest break will be scheduled during the class.  c. Nurses will monitor patient fatigue throughout the class, observing physical signs, monitoring posture, and checking in with patients about their need for rest.  A multifaceted approach to fatigue management will be implemented in the dance class.  a. If a patient is fatigued prior to class, their participation will be reassessed.  b. A rest break will be scheduled during the class.  c. Nurses will monitor patient fatigue throughout the class, observing physical signs, monitoring posture, and checking in with patients about their need for rest.  Quantitative data on how dance impacts fatigue is needed. Therefore, fatigue will be assessed quantitatively before and after dance sessions in select patients to inform future program parameters, including intensity and duration. |
|  |  | Quantitative data on how dance impacts fatigue is needed. Therefore, fatigue will be assessed quantitatively before and after dance sessions in select patients to inform future program parameters, including intensity and duration. |
| **To use improvisation or not** |  |  |
|  | Improvisation is common in dance, however, dance health research suggests it can lead to class delays and unpredictable movement patterns. | Through QB/GU workshops it was decided that a method is needed to enable patient agency and movement creation while minimizing the risk of harm or disruption. Therefore:  a. Dance classes will be facilitated so that patients do not instruct other patients (no mirroring or echoing of patient created movements).  b. Parameters will be set to limit the improvised movement, such as restrict range of motion to a certain body part (e.g., arm or hand), or provide focused movement prompts.  c. The use of improvisation will be adjusted as needed, such as having patients describe favorite activities, with dance teachers creating movements to match. |
|  | Allied health consultation and dance health literature and experience from the teaching team suggest that the use of improvisation poses potential challenges due to limitations in abstract thought. | Through QB/GU workshops it was decided that dance teachers will trial various facilitation strategies to identify the most beneficial approach. |

**Table S3**. The DAN-CER program mapped to the TIDieR [1] checklist.

| Item | Description |
| --- | --- |
|  |  |
| **Brief Name** |  |
|  | Dance as an Adjunct therapy for Neurological rehabilitation – Creative Enrichment for Recovery  (DAN-CER). |
| **Why** |  |
|  | Rationale for the intervention: Patients in inpatient neurological rehabilitation desire meaningful social activities during the time between therapy sessions. Well-researched elements such as synchronised movements and peer collaboration, provides an ideal foundation for an adapted dance recreation program aimed at improving patients’ sense of social connection, affect, and wellbeing.  Goal: The goal of the program is to foster interpersonal interaction, agency, and enjoyment through collaboration, movement and music. |
| **What** |  |
|  | What materials: Music is played on a portable speaker, controlled with a smart watch. Assistance bars or sturdy tables are available for balance for patients who require them, depending on location. All participants wear appropriate footwear. Patients participating in a seated manner are provided with appropriately supportive chairs. Some participants use standard wheelchairs or tilt in space wheelchairs. For ambulant participants, some can walk independently, while others require assistance from the RAP staff to walk. This is identified during the screening process by the Rehabilitation Medical Director.  What procedures: Each class accommodates a maximum of six patients. Two female teaching artists, two RAP nurses, and an additional workload nurse (available when necessary) assists the session. Two wardsmen are available to assist with patient transportation. Therapists are invited to attend the sessions. On Monday afternoons, the class plan is sent to the medical team for distribution with allied health staff to screen. Staff ensure that patients perform and practice any movements of concern during the week to confirm they are safe to perform those movements. On Fridays, the medical team completes a list of eligible patients for each Saturday’s class. This list, along with any important information regarding patient participation, is handed to RAP nurses on the day of the class. During the class, a formal break is provided midway, and staff encourage patients to take individual breaks as needed. |
|  | What dance activities: The program includes a mix of theoretically derived and evidence-based dance techniques and concepts. Movements are predominantly mindful based movements and simple rhythmic movements. Each week features a theme (used twice in succession) to guide music selection, movement choice, and imagery. The themes are 1. *50s/60s,* 2. *70s/80s* and 3. *Seasons.*  The classes consist of the following sections:  Unless stated otherwise, participants are positioned in a large semi-circle, with RAP nurses dispersed between patients.   1. Mindful warm up: Exercises focus on breath, body awareness, posture, and exploring how the body feels.   *Example:* Using breath to transition from slumped to upright posture, or using hand breathing to reach out with arm/s.  *Music:* Instrumental music to aid concentration.   1. Rhythm section: Body percussion involving different rhythms.   *Example*: Tapping (either hand, or foot, one or both), clapping (modification for hemiparesis or limb difference involves tapping on an opposite limb), or tapping only on first beat of the bar.  *Music:* Theme related music (e.g., 50s/60s), 130 – 160 bpm.   1. Moving arms and legs: (x 2 exercises): Simple fun movements with small to moderate range of motion.   *Example: S*houlder rolls, windscreen wiper hands, shoulder shrugs, marching to the beat.  *Modifications:* a.) seated hip flexion, b.) alternating foot taps.  *Music:* Theme related music, 120 – 140 bpm  *Positioning: S*eated patient dancers in two lines (staggered to be able to see), standing patients at the parallel therapy bars.   1. Mindful movement fatigue buster: Tai chi / Pilates based movements focusing on breath and body awareness.   *Example:* Gentle movements based on imagery of hands skimming on water, stroking the waterfall, or hands holding an expanding energy ball with the breath.  *Music:* 60 bpm to induce relaxation.   1. Collaborative creation: *Example*: Patient dancers share their favourite move/memory from the theme of the week (e.g., 50s/60s). Movements are joined together to form a movement phrase for everyone to complete. The patient dancers first demonstrate or explain their movement to the teaching artist or RAP nurse, who moderates the movement as necessary.   *Music*: Choice of two songs selected by patients.   1. QB Repertoire: Patient dancers experience modified repertoire movements from ballets previously performed by QB.   *Example:* Coppelia – Mazurka  *Movement examples:* Small leg extension with flexed foot (to mimic kicking a ball), closed fist and raised arm to imitate victory.   1. Mindful cool down: Relaxation style movements to aid recovery.   *Movement example:* Painting the air, pointing and flexing toes. |
| **Who provided** (Will provide) |  |
|  | Who will provide: The classes are instructed by one qualified dance teacher (DP) with experience working with diverse populations in an exercise physiology clinic, specialising in mindful movement and exercise prescription. Additionally, one teaching artist from QB, with experience running dance health classes for a range of individuals including those with neurological disorders, nursing home residents, will assist with the sessions. Both teachers were instrumental in the creation of the program. |
| **How** |  |
|  | Delivery: Classes are delivered in a group setting, face to face. All exercises are conducted with the dance teachers modelling the exercises and providing cues, while the RAP staff assist and monitor patients’ wellbeing, also participating when able.  Teaching: Cuing: Each exercise is introduced with a brief demonstration of the upcoming movements. Preliminary cues are directed to the RAP staff, such as: “This exercise requires you to act as a Movement Monitor. If a patient is moving outside of what is safe, please provide corrective instruction.” This is followed by a cue to patients that is clear and simple outlining the purpose of the exercise, and any crucial safety instructions. For example, “This exercise is all about choice, creativity and connection. We will all stay seated.”  During the exercises, cuing follows an aerobics-style approach for simple changes. Movement changes are cued in advance, through a countdown of repetitions, or upon a distinct music change (e.g., chorus). For more complex changes, the verbal cue “stop” accompanied by a hand gesture is used. The movement is first demonstrated with the verbal cue “watch me”, followed by “now join me.”  Corrections essential for safety are delivered using active language (instructing participants on what they need to do, rather than pointing out what they are doing incorrectly). For example, if a participant is leaning too far away from their body, dance teachers would say “small movements only”. |
| **Where** |  |
|  | DAN-CER is conducted in a portion of the rehabilitation therapy room, with the available space measuring 9 x 4 metres, or another open space convenient to the hospital. |
| **When and how much** |  |
|  | The dance classes are held once weekly, on Saturday mornings at 10:00 am, with each session lasting a maximum of 60 min. The program runs for 6 weeks beginning in mid-October. The overall intensity of the classes is light to moderate. |
| **Tailoring** |  |
|  | 1. The program includes inbuilt tailoring to manage fall risk. Clinicians assess patients’ ability to participate in the upcoming weekend’s dance class based on the class run sheet provided to them. Fluctuating health or fatigue levels are also considered, and participation levels are modified accordingly. As a result, the majority of the class is conducted in a seated position, with alternatives for two exercises allowing movements to be performed either seated or standing with support or standing with the nurse nearby (if able and cleared by allied health staff). For example, moving in time to the beat may involve seated foot tapping, seated marching, standing marching holding onto a rail, or standing marching unassisted. 2. Duration of each exercise and the class length are moderated by dance teachers in situ. For example, if progress stagnates during the movement creation section, the duration or structure of the activity will be adjusted. Similarly, if nurses raise concern about fatigue, the session length will be shortened by removing one or two exercises. 3. The speed of exercises is tailored in situ (some patients may need to perform movements at half speed). This is enacted by one teaching artist performing the exercise at a reduced speed for the patient to mirror. 4. Formation: Participants in Tilt in space wheelchairs are positioned in the most effective locations (e.g., to the side, but positioned diagonally) to ensure visibility of the teachers and nurses. This positioning is adapted as necessary. |
| **Modifications, how well planned, and how well actual** |  |
|  | Modification at the intervention level, as well as adherence and fidelity, will be reported in future publications. |

**Table S4**. Data sources for the primary and secondary outcomes of the DAN-CER pilot

|  | Quantitative data | Qualitative data | | |
| --- | --- | --- | --- | --- |
| **Domain** |  | Operational field notes | Patient semi-structured interview’s | Staff semi-structured interview’s |
|  |  |  |  |  |
| **Recruitment** | - Number of participants interested and enrolled over the program. - Number of participants interested that did not pass the UBACC cognitive screener. - *Target: 6 per week interested.* | - Barriers and facilitators to recruitment collected from field notes. | na | na |
| **Attendance and retention** | - Attendance rates. - *Target: Patients participate in 80 % of each class and 80 % of the classes available to them.* - *Target attendance rates: 4 participants per week.* - Number of participants who dropped out due to health decline or dislike calculated as a proportion of the total number recruited. | - Field notes of reasons for non-attendance e.g., family visitor, pain, sickness. - Field notes of challenges to implementation e.g., noise, crowding, patients being late. - Reasons for drop out e.g., medical episode. | - Themes or notes of barriers and facilitators to delivering the intervention e.g., facilitatory themes such as the classes being enjoyable, and engaging and barrier themes such as fatigue, or pain. | - Themes or notes of barriers and facilitators to delivering the intervention e.g. facilitators such as it being conducted at a manageable time, and the use of ward staff or barrier themes or notes such as understaffing. |
| **Safety** | - Number of adverse events e.g., falls (warrants reporting to ethics). | - Any observable minor harms noted by any stakeholder (dance teacher, on shift nursing staff). - Modifications to class plan requested from allied health staff. - Modifications of proposed dance program from teacher notes e.g., changes to class structure or facilitation techniques for safety. | - Themes or notes regarding safety concerns. | - Themes or notes regarding safety concerns. |
| **Acceptability** | Na. | - Any field notes of comments raised during the class. - Field notes of patient comments during the week regarding acceptability. - Field notes representing unresolved barriers. - Field notes from the multidisciplinary team indicating that the exercises conflict with those recommended by rehabilitation clinicians. | - Themes of perceived benefits e.g., physical, cognitive, or social. - Themes of experiences. - Themes indicating non acceptability i.e.., difficulty participating, discomfort, frustration, excessive fatigue, or psychological distress. - Themes of ongoing commitment e.g., would like to see it continue. | - Themes indicating non acceptability e.g., excessive nursing staff or allied health burden. - Themes of observations. - Themes of experiences. - Themes of ongoing commitment e.g., would like to see it continue. - Themes of perceived benefits e.g., physical, cognitive, or social. |
| **Single case design pilot**  Case study patients only. | - Pre and post program Wellbeing. - Pre and post program Depression. - Pre and post program Anxiety. - Pre and post program Stress. - Pre and post session affect. - *Target: RCI* - Missing data points. - How many patients had a full SCD data set. - Pre and post session fatigue. | - Notes on operational/logistical aspects of the study e.g., environmental, patient stay estimations, staffing challenges, sickness. - Patient comments on the measures or methods being used. - Notes on reasons for missing data: e.g., Patient incoherence, pain, medical interruption, medical episode, fatigue, or human error. | - Themes of any perceived benefit to wellbeing. - If so, themes of what may have been the driving force of outcomes   e.g., movement opportunity, music, or socialization. | - Themes of any perceived benefit to wellbeing. - If so, themes of what may have been the driving force of outcomes.   e.g., movement opportunity, music, or socialization.   - Themes in what staff observed. |

## Reference

1. Hoffmann TC, Glasziou PP, Boutron I, et al. Better reporting of interventions: template for intervention description and replication (TIDieR) checklist and guide. BMJ. 2014;348:1-12. DOI:10.1136/bmj.g1687
